# Development and Validation of the Knowledge and Attitude Scale Toward COVID-19 Pandemic Breaking Transmission Chain (KA-C) Among Iranian Population

**DOI:** 10.3389/fpubh.2021.627013

**Published:** 2021-02-17

**Authors:** Mahdi Abounoori, Mohammad Moein Maddah, Hamid Sharif Nia, Pardis Rahmatpour, Shaghayegh Khosravifar, Mohammad SamadiKouchaksaraei, Shahrzad Khosravifar

**Affiliations:** ^1^Student Research Committee, School of Medicine, Mazandaran University of Medical Sciences, Sari, Iran; ^2^School of Nursing and Midwifery Amol, Mazandaran University of Medical Sciences, Sari, Iran; ^3^School of Nursing and Midwifery, Iran University of Medical Sciences, Tehran, Iran; ^4^Department of Psychiatry, School of Medicine, Isfahan University of Medical Sciences, Isfahan, Iran; ^5^School of Nursing and Midwifery, Mazandaran University of Medical Sciences, Sari, Iran; ^6^Department of Medicine, Aliasghar Children Hospital, Iran University of Medical Science, Tehran, Iran

**Keywords:** attitude, knowledge, COVID-19, pandemic, populations

## Abstract

**Objectives:** We aimed to develop a scale and evaluate this scale's validity and reliability to measure factors affecting people's knowledge and attitudes toward the pandemic breaking transmission chain.

**Methods:** This exploratory mixed-method study was carried out in two phases: (1) item generation using literature reviews and interviews and, (2) item reduction by psychometric assessments of the developed scale. The face, content, construct (exploratory and confirmatory factor analysis), convergent, and discriminant validity of the scale were assessed in the Iranian population (*n* = 500) from March to June 2020. The Composite Reliability (CR) and the internal consistency correlation coefficient were estimated.

**Results:** The Knowledge and Attitude Scale Toward COVID-19 Pandemic Breaking Transmission Chain (KA-C) among the Iranian population included 18 items. Two factors with a whole variance of 66.05% were identified by exploratory factor analysis. Factors were labeled as “health literacy” and “home health empowerment.” The confirmatory factor analysis showed the goodness of fit. The CR of the scale for first and second factors were 0.965 and 0.833 receptively. The scale's internal consistency correlation coefficient was acceptable (Cronbach's alpha = 0.960 and 0.823, average interitem correlation = 0.643 and 0.635, McDonald's omega = 0.963 and 0.829, for the first and second factor, receptively).

**Conclusion:** The KA-C scale can be exerted to screen the people's knowledge and attitude about the COVID-19 pandemic breaking the transmission chain as a valid and reliable scale for further policymaking, health care providers, and for a multi-dimensional psychosocial assessment of the pandemic period.

## Introduction

The world has been facing a new challenge in the past several last months. The new coronavirus, SARS-CoV-2, first emerged in Wuhan, Hubei province China, and has since then turned into a global pandemic (1). As a first response to control the spread of this new disease, travel bans and quarantine were introduced in many countries (2). Several international borders have been shut, many businesses have been closed, and billions of people were quarantined or isolated in their own homes. Since China was the first place of the novel coronavirus emergence, the government mandated a policy to quarantine Wuhan city on January 23, 2020. Other governments have also paid attention to this policy's effectiveness and have implemented it in their own countries (3). Reports of Chinas experience in public health control have shown that the number of people infected with COVID-19 and the peak of the spread was effectively reduced by reducing latent individuals' interaction after quarantine and isolation. As a result, citizens' communication restriction policies, such as quarantine and isolation, are necessary for controlling the spread of COVID-19 (4). On the other hand, the World Health Organization (WHO) (5) and the Centers for Disease Control and Prevention (CDC) have declared social distance, hand, and respiratory hygiene as the best and easiest strategies to control and prevent infection (6).

COVID-19 can be transmitted through human-to-human contact and indirect contact with contaminated objects. COVID-19 can be transmitted through body fluid droplets from the mouth or nose, spreading when a person with COVID-19 coughs, sneezes, and talks. Droplets typically cannot transverse more than six feet (almost 2 m). COVID-19 remains intact and contagious in droplets and can be suspended in the air for up to three hour. Additionally, contaminated droplets can settle on plastic, stainless steel, copper, and cardboard objects. A person can become infected if they touch the surface contaminated with COVID-19 objects and then contact mucous membranes such as the eyes, nose, or mouth. Therefore, health experts advise frequent hand-washing with soap and water (7). It seems to modify these variables and plays a key role in preventing and breaking the transmission chain.

People's awareness of the strategies mentioned is also crucial to breaking the transmission and contamination chain. During public health emergencies, official sources (like newspapers, press releases, and educational messages) and unofficial sources (like social media, online reviews, views of family, and peers) play a major role in people's awareness (8, 9). Therefore, people's awareness about preventing new infections relies on these information sources (10). The role of official and unauthorized sources of information on situational awareness (perceived understanding) is the focal point of recent studies. Eventually, these sources have an essential impact on people's protective actions during a public health emergency as shown by the adoption of protective behaviors (social distancing). It has also been mentioned that situational awareness could affect people's adoption of social distancing (8, 11). However, there has been a greater emphasis on formal information source's impact on people's protective behavior adoption (11). In a methodological framework focused on a narrative analysis of data collected from the 2003 outbreak of the SARS virus, it is stated that the decisions and actions of people during an outbreak are mainly based on the cognitive representations of events of individuals, which are in turn influenced by prior knowledge and new information (12).

In Iran, after the first case of COVID-19 was registered on February 19, 2020, attempts have been developed to shut down religious congregations, schools, and colleges, as well as non-essential businesses. Efforts have been directed toward continuing education courses online. All cinemas, theaters, sporting activities, and gymnasiums were closed. Car and real estate businesses were also closed and hotels and accommodation centers received nearly zero guests. It was eventually declared that no place of business was safe from COVID-19 (13). During this situation, public health was effected by misinformation on social media, which promoted invalid advice like consuming alcohol to prevent the virus. Also, breaking quarantine, trusting invalid sources of information, and not adhering to official advice led Iran to experience the second wave of disease. Attitudes during crisis is therefore essential in addition to knowledge. Incorrect attitudes such as abandoning responsibilities and incorrect policies deepen this crisis (5, 13–15). Still, after the opening of activities under health protocols in Iran, and due to poor attitude and knowledge, we saw increased reports of death in Iran and a consequent third wave of the disease (5). By studying knowledge and attitudes, information on what is known, believed, and practiced by the general population can be collected. Such information is necessary because unclear information and negative attitudes toward viral diseases among the community may lead to distress, panic, and other psychiatric disorders or may exacerbate preexisting ones. In this time of crisis, research on the general population's knowledge and attitudes play a pivotal role in understanding the public's level of awareness about the knowledge, attitudes, and best practices toward COVID-19 (7). This study aimed to develop and assess the psychometric characteristics of the scale, which assesses the Iranian people's awareness and attitudes toward the COVID-19 pandemic and how to break the transmission chain as well as how these attitudes are practiced. The designed scale focuses on people's understanding and familiarity with health-protective behaviors and how the outcomes of this information will impact their decisions on virus transmission blockade collaboration. It is worth mentioning that it is the first time that experts have developed a scale to yield objective scores in this regard.

## Methods

This exploratory mixed-method study was conducted in two phases, including item generation and item reduction. Literature reviews and interviews were conducted in the item generation phase, and psychometric assessment of the scale was conducted in the item reduction phase (16).

### Item Generation

#### Literature Review

In the literature review, the identification of terms attributed to knowledge and attitude was reviewed using WHO, CDC, and Johns Hopkins COVID-19 Hub websites from December 2019 until May 2020. The terms that have been searched in different databases (e.g., Science Direct, Scopus, PubMed, Web of Science and Persian databases such as Magiran, SID, and IranMedex) were “COVID-19,” “awareness,” “health protective behaviors,” “animal hygiene” and “social distancing.” The inclusion criteria were defined as any type of article and document related to COVID-19 prevention which were published between December 2019 and May 2020. Fifteen studies (13 English and two in Persian) were selected. After reviewing abstracts and full texts and excluding duplicate articles, seven articles were included in the study. Articles were mostly about the factors influencing the transmission and prevention of the virus such as correct and incorrect behaviors, knowledge, attitude, and beliefs related to the spread of the virus in the general population. Two independent researchers (MA and MM) performed article screening. Through this process, if there was a disagreement, a third reviewer (SK) made the final decision. According to the steps proposed by Graneheim and Lundman (17), the conventional content analysis method was used to extract initial codes from seven included studies that were in line with the study's objectives.

#### Interview

Twenty semi-structured face-to-face interviews were organized to achieve a better overview of the concept of attitude and knowledge. Using purposeful sampling, the participants (both healthy and sick people) were selected from the general population. On average, individual interviews took around 30 min. The interview questions were focused on the following sections:

We want to know and assess what factors are involved in improving the contributor's (you) collaboration to obey instructions published from health organizations?
Also, to assess people's tendency to consume formal or informal sources of information, we asked how patients get their information about COVID-19 or how they prepare themselves for protective behaviors?
The interviews were recorded and transcribed. The texts of the interviews were analyzed with a directed content analysis method using MAXQDA software Ver.10.

### Item Reduction

The components of people's awareness about breaking the transmission chain extracted in the first and second phases of item generation have emerged.

Based on extracted codes, categories, and subcategories, an item pool with 23 items was prepared. Items were reviewed by researchers several times, and then some were removed and modified. Finally, an initial format of the knowledge and attitude toward COVID-19 (KA-C) scale with 23 items was prepared. The response options were based on a five-point Likert scale: “I completely agree, I agree, I don't agree nor disagree, I disagree, and I completely disagree.”

#### Face Validity

Quantitative and qualitative methods were used to determine the face validity of KA-C (18). For qualitative face validity, face-to-face interviews were conducted by convenience sampling 10 participants, and they were asked to indicate whether they felt difficulty or ambiguity in answering the item. The participants used for face validity included three adolescents aged between 12 and 19, four adults aged between 20 and 65, and three geriatric participants aged over 65 years. Then, according to the views of the research team and participant recommendations, the items were edited. In the quantitative face validity, the impact score of each item was calculated. Ten participants aged between 12 and 19 years rated items about their clarity and indicated one of the following answers for each item: “it is completely important, it is important, it is almost important, it is a little important, it is not important at all.” The scores ranged between one and five, in which the score of one represents the lowest value and the score of five the highest. The impact Score = Frequency (%) × Significance (19). The impact score was deemed to be > 1.5 (20).

#### Content Validity

Ten experts with expertise and experience in psychometric assessment (*N* = 2), psychology (*N* = 2), epidemiology (*N* = 2), and disaster management (*N* = 4) were requested to apply their analysis of the scale in terms of grammar, phrasing, item allocation, and scaling to assess the qualitative content validity. Their recommendations were considered in the scale. The quantitative content validity was examined by calculating the content validity ratio (CVR) and content validity index (CVI). The same 10 experts then specified each item's necessity for the CVR evaluation. They scored each item as 1 = unnecessary, 2 = somewhat necessary, or 3 = necessary. Based on the Lawshe formula (1975), when 10 experts' opinions are considered, the minimum acceptable CVR is 0.64 (21). For evaluating the relevancy of the scale, CVI was employed and ranged from one = not relevant to four = relevant. The mean of CVI scores was taken into account to measure the scale content (I-CVI average for all items on the scale). A score of 0.9 is considered excellent, and 0.8 is considered appropriate (22). Based on the above considerations, two items were removed (due to CVR and CVI of lower than 0.64 and 0.8), and the items of KA-C were reduced to 21 items.

#### Participants and Setting

Cyberspace and social media (WhatsApp, Telegram, Instagram, LinkedIn) were used for data gathering. The 21-item scale was prepared using Google Forms at https://forms.gle/4qngsnDXFnkAcJdj7. (in the Persian language). Google Forms is an online questionnaire creation software. Considering 10 samples for each item (23), 210 individuals (21 items × 10) were considered for each section of the factor analysis (exploratory and confirmatory). Literate people of any age who were willing to participate in the study formed the inclusion criteria. Overall, 500 individuals participated in construct validity; 250 for exploratory factor analysis (EFA) and 250 for confirmatory factor analysis (CFA).

#### Construct Validity

In this study, the EFA was evaluated through maximum likelihood and Promax rotation. Bartlett's sphericity test and the Kaiser-Meyer-Olkin (KMO) index were used to determine the sample's adequacy for performing the factor analysis. A KMO of higher than 0.9 indicated an adequate sample (24). The factor extraction was based on an absolute factor loading value that should be > 0.3, eigenvalues > 1, communalities > 0.2, and scree plots.

The factor structure obtained from the EFA was examined and established by performing CFA. Any item that did not reach a coefficient of correlation higher than 0.5 was removed from the scale at this phase (25). The utilized fit indices in the study included Chi-square (χ^2^), Degree of Freedom (DF), Parsimonious Comparative Fit Index (PCFI) >0.5, Parsimonious Normed Fit Index (PNFI) >0.5, Minimum Discrepancy Function divided by Degrees of Freedom (CMIN/DF) (<3 good, <5 acceptable), Comparative Fit Index (CFI) > 0.90, Incremental Fit Index (IFI) > 0.90, Tucker–Lewis index > 0.90, and Root Mean Square Error of Approximation (RMSEA) <0.08 (26). To evaluate the construct validity, SPSS version 26 was used for exploratory factor analysis and confirmatory factor analysis (CFA), evaluated using Analysis of Moment Structure software (AMOS) version 24.

#### Convergent and Divergent Validity

Based on the Fornell (27) criteria, the convergent and divergent validity of KA-C was assessed using Average Variance Extracted (AVE) (28), Maximum Shared Squared Variance (MSV), and Composite Reliability (CR). To find the convergent validity, it must be AVE >0.5 and CR > AVE, and it should be AVE > MSV to establish the divergent validity (29).

#### Reliability

The internal consistency of the current scale was calculated by Cronbach's alpha, Average Interitem Correlation (AIC), and McDonald's omega (30). The acceptable minimal reliabilities of Cronbach's alpha and McDonald's omega are 0.7 (31), and for AIC, the acceptable value is 0.2–0.4 (32). A CR value > 0.7 was regarded as the desirable reliability (29).

### Ethical Considerations

The protocol of this study was approved by the Ethics Committee of the University of Medical Sciences of Mazandaran (IR.MAZUMS.REC.1399.596). All individuals voluntarily participated in this study. The names of the contributors were not mentioned in the report, and codes were used in the interview texts instead of their names. The declarations of the participants were not shared with other members of the research team to protect the confidentiality of the information. Furthermore, in the initial set of data gathering, participants were ensured that all answers will remain confidential and that involvement in the study is voluntary.

## Results

A total of 500 individuals participated in this study. The majority of them (65.2%) were female. In the age group, most of the participants were 21–40 years old. 55.6% of the participants were educated in the field of experimental science, with only a few (39.2%) being educated in the field of medical and paramedical sciences. The remaining participants (60.8%) were educated in other fields (see Table 1).

**Table 1:** Demographic characteristics of study participants (*n* = 500).

**Variables**		***N* (%)**	**Variables**		***N* (%)**
Age (years)	0–20	84 (16.8)	Field of study	Experimental	278 (55.6)
	21–40	337 (67.4)		Mathematics	88 (17.6)
	41–60	67 (13.4)		Humanities	125 (25)
	> 60	12 (2.4)		Theological	9 (1.8)
Gender	Male	174 (34.8)	Education in	Medicine and paramedicine	196 (39.2)
	Female	326 (65.2)		Others	304 (60.8)
Educational degree	Elementary	5 (1)	Householder	Yes	82 (16.4)
	Middle school	11 (2.2)		No	418 (83.6)
	Diploma	104 (20.8)	Employment	Employed	93 (18.6)
	Associate degree	42 (8.4)		Unemployed	93 (18.6)
	Bachelor's	194 (38.8)		Self-employed	99 (19.8)
	Master's	55 (11)		Soldier	3 (0.6)
	PhD	20 (4)		Medical and paramedical student	125 (25)
	General physician	33 (6.6)		Other Fields student	87 (17.4)
	Specialist physician	22 (4.4)			
	Subspecialist physician	14 (2.8)			

*Development and validation of knowledge and attitude scale toward COVID-19 (KA-C) pandemic among Iranian population, Iran, 2020*.

After performing face and content validity, the 21-item scale entered the construct validity step. In MLEFA, the KMO test value was 0.960, and the Bartlett's test value was 8,513.860 (*P* < 0.001).

Two factors were extracted (18 items) and named as “health literacy” (15 items) and “home health empowerment” (three items). These two factors explained 66.05% of the variance (see Table 2).

**Table 2:** The two factors of the Knowledge and Attitude Scale Toward COVID-19 (KA-C) pandemic among Iranian population and their factor loadings (*n* = 250).

**Factors**	**Items**	**Factor** **loading**	***h*^2^**	**λ**	**% variance**
Health literacy	Q17. During the COVID-19 outbreak, I disinfect food that prepares from outside, such as fruits and vegetables and packaged foods.	0.909	0.860	8.53	47.42
	Q16. During the COVID-19 outbreak, I frequently disinfect surfaces and objects.	0.887	0.814		
	Q18. During the COVID-19 outbreak, with those around me, I observe the maximum appropriate distance (4 meters).	0.864	0.757		
	Q11. During the COVID-19 outbreak, I will follow the quarantine or social distancing until the Ministry of Health notifies it.	0.855	0.703		
	Q15. During the COVID-19 outbreak, I use a mask Outside the house.	0.843	0.741		
	Q14. During the COVID-19 outbreak, If I have sneezing or coughing, I use a handkerchief, and then I throw it in the trash.	0.836	0.737		
	Q7. During COVID-19 outbreaks, I frequently wash my hands with soap and water.	0.828	0.766		
	Q10. During the outbreak of corona, I avoid touching my face, eyes, nose, and mouth without proper washing.	0.812	0.703		
	Q9. During the COVID-19 outbreak, I prefer to wash my hands with soap and water over alcohol and other antiseptic solution.	0.795	0.706		
	Q8. During COVID-19 outbreaks, I frequently wash my hands with alcohol and antiseptic solutions.	0.757	0.577		
	Q21. During the COVID-19 outbreak, If I have a problem with my pet's health, I consult a veterinarian.	0.743	0.599		
	Q13. If I feel any warning and emergency alarm signs of coronavirus by myself (e.g., respiratory distress, constant pain or pressure in the chest, dizziness, lips or bruised face, etc.), I rush to the medical center.	0.709	0.570		
	Q12. If I feel I have COVID-19, I will stay home.	0.706	0.648		
	Q19. I have a pet, and during the COVID-19 outbreak, after contact with animals, pet food, pet collars, and other items, I wash my hands with soap and water or other disinfectants.	0.695	0.514		
	Q1. I get my information about COVID-19 from the official pages of the WHO.	0.357	0.226		
Home health empowerment	Q5. During the COVID-19 outbreak, I accurately monitor high-risk family members, such as those with chronic illnesses.	0.926	0.869	3.35	18.62
	Q4. During the outbreak of COVID-19, I precisely monitor high-risk family members, such as the elderly.	0.922	0.841		
	Q6. I have a room at home for the COVID-19 infected person caring.	0.396	0.358		

**Figure 1:**
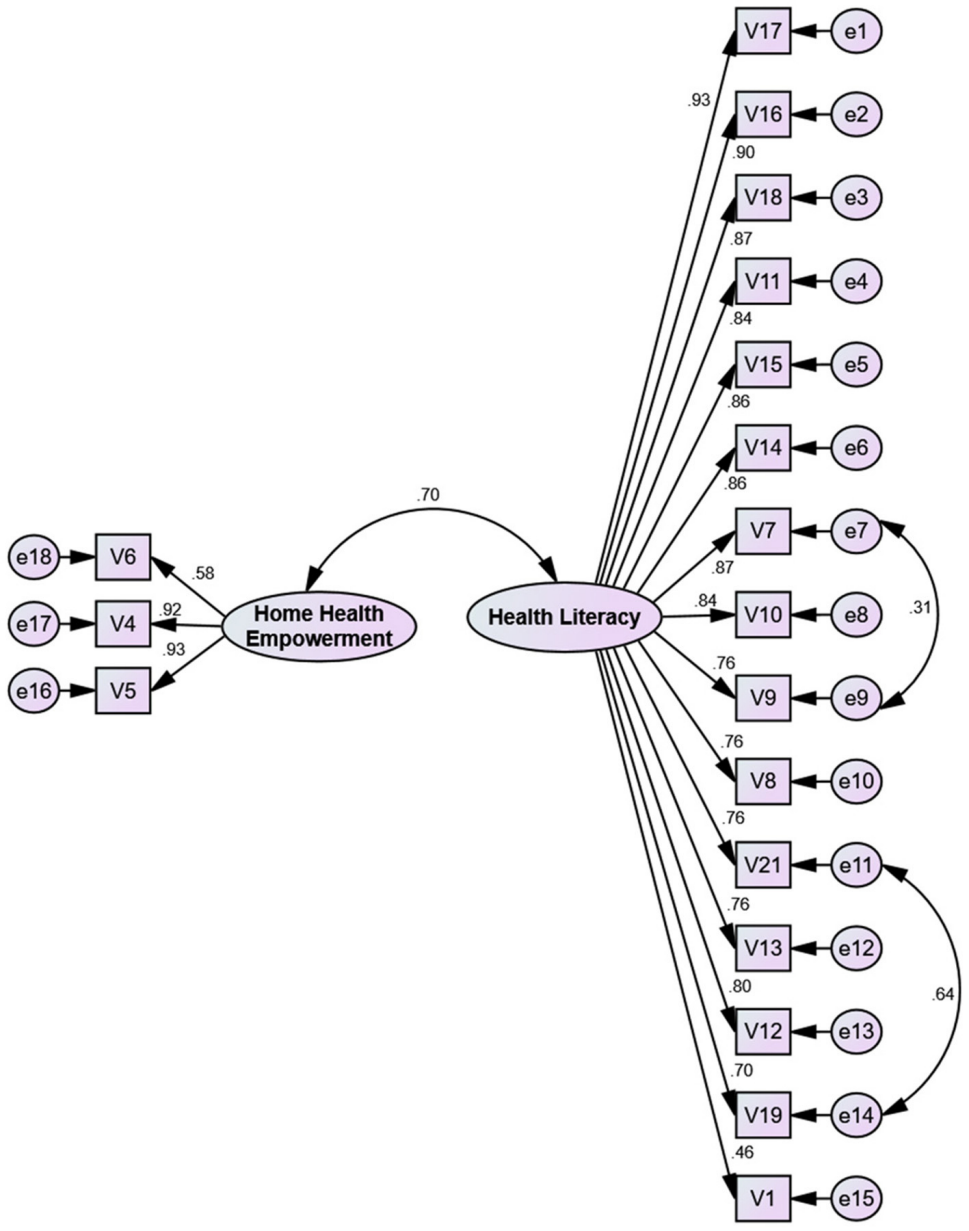
The Knowledge and Attitude Scale Toward COVID-19 (KA-C) pandemic among the Iranian population construct: the modified first-order confirmatory factor analysis (*n* = 250).

In the confirmatory factor analysis, after modifying the model, the Chi-square model fit index was evaluated as 373.160 (DF = 129, *P* < 0.001) and CMIN/DF = 2.893. Then, other model fit indices were calculated, and the good fit of the final model validated those values (see Table 3, Figure 1).

**Table 3:** Fit indices of the confirmatory factor analysis (CFA) of the Knowledge and Attitude Scale Toward COVID-19 (KA-C) pandemic among Iranian population (*n* = 250).

**Indices**	**χ**	**DF**	***P*-value**	**CMIN/DF**	**RMSEA [CI90%]**	**PNFI**	**PCFI**	**TLI**	**IFI**	**CFI**
First-order CFA after structure modification	373.160	129	<0.001	2.893	0.062 [0.054 to 0.069]	0.807	0.819	0.966	0.971	0.971
Second-order CFA after structure modification	373.387	130	<0.001	2.872	0.061 [0.054 to 0.069]	0.813	0.825	0.966	0.971	0.971

*DF, Degree of freedom; PCFI, Parsimonious Comparative Fit Index; PNFI, Parsimonious Normed Fit Index; CMIN/DF, Minimum Discrepancy Function divided by Degrees of Freedom; RMSEA, Root Mean Square Error of Approximation; TLI, Tuker-Lewis Index; CFI, Comparative Fit Index; IFI, Incremental Fit Index*.

**Figure 2:**
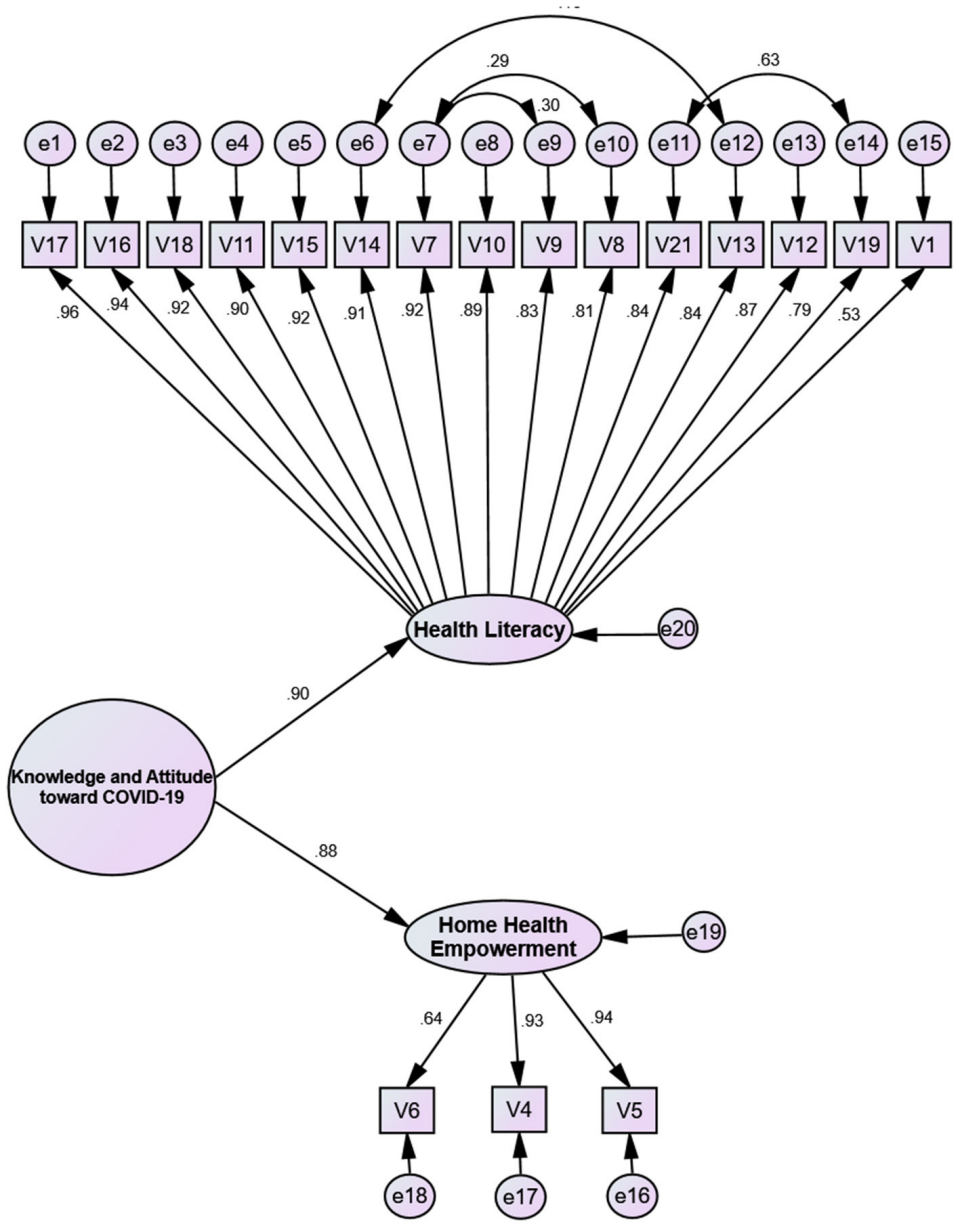
The Knowledge and Attitude Scale Toward COVID-19 (KA-C) pandemic among the Iranian population construct: the modified second-order confirmatory factor analysis (*n* = 250).

The AVE, MSV, and CR results confirmed the convergent validity; however, the discriminant validity is not confirmed (see Table 4). The MSV of the two factors was not less than AVE. This confirmed that the extracted factors are not separate from each other and that running a second-order CFA is required (see Figure 2 and Table 3).

**Table 4:** The indices of the convergent, discriminant validity, and internal consistency of the Knowledge and Attitude Scale Toward COVID-19 (KA-C) pandemic among Iranian population.

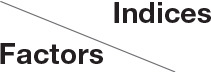	**AVE**	**MSV**	**CR**	**Alpha (CI95%)**	**AIC**	**Omega**
Health literacy	0.64	0.70	0.965	0.960 (0.958 to 0.968)	0.643	0.963
Home health empowerment	0.68	0.70	0.833	0.823 (0.803 to 0.855)	0.635	0.829

*AVE, Average Variance Extracted; MSV, Maximum Shared Squared Variance; CR, Composite Reliability; Alpha, Cronbach's alpha; AIC, Average inter-item Correlation; Omega, McDonald's omega coefficient*.

Internal consistency of the scale revealed that the Cronbach's alpha, McDonald's omega, and AIC of two factors were acceptable. The CR of factors also showed that there was good reliability (see Table 4).

## Discussion

This study was intended to develop and provide a psychometric analysis of the people's knowledge and attitude toward the COVID19 (KA-C) pandemic breaking the transmission chain among the Iranian population between March to June 2020. The scale was developed using a comprehensive review of the literature and interviews, expert panel reviews to prospect content and face validity, EFA, and Cronbach's alpha coefficients to descry internal consistency and reliability. With 18 items, the KA-C focused on the profound impact of knowledge and attitude in COVID-19 prevention.

People's knowledge and attitudes are important in public health, especially in pandemic disease control strategies. Knowledge (cognitive behavior) impresses on attitude (emotional behavior), and attitude impresses on health behavior. So, when an outbreak happens, controlling disease spread is linked to disease-related literacy and people's attitudes (33). Thus, studies on people's knowledge and attitudes in the spread of disease are important and can change behaviors. It will help to recognize the existing gaps in information about COVID-19 by deciphering the degree of knowledge and attitudes toward this unprecedented pandemic (34). Scales must be used to fill this gap and encourage a sound awareness of COVID-19, which would aid in disease control. Behavioral improvements and positive attitudes rely primarily on the level of COVID-19 expectations and knowledge of preventive activities (35).

The EFA indicated that the scale has two factors. The first factor is “health literacy” (15 items), and the second one is “home health empowerment” (three items). The “health literacy” can reflect the knowledge of participants, which can directly affect their attitudes. The first step in guiding future efforts in the educational or policymaking process, which has been shown to impact future actions, is to determine knowledge of precautionary actions for contracting the disease (36). Proper knowledge about the pandemic mainly originates from reliable formal sources of information. It is essential to strengthen knowledge among populations *via* health education because COVID-19 has been declared a pandemic. Therefore, an improved understanding will affect attitudes and practices toward COVID-19 (7). The “home health empowerment” reflects people's negative and positive attitudes on how they get ready to practice protecting the sick and healthy from COVID-19. As mentioned earlier, a lack of understanding also contributes to an unconcerned attitude that can negatively affect overcoming these challenges (37).

Since this disease is new and, despite the extensive research done in the study of different ways of its transmission, we see that observers still sometimes suffer from this disease. Therefore, the scale items are prepared to examine how people are informed by news related to this disease, how this disease could be transmitted, and how they can prevent themselves and others from becoming infected with this disease. The items were designed so that factors such as age and gender do not significantly impact how to answer. Still, factors such as cultural and economic features in the answers may be effective (38).

In this study, based on CVI and CVR, two items were omitted from the scale.

From the results, the two factors' variance is 66.05%, which is acceptable, considering that this value is above 50%. It is also mentioned that if a factor above 60% achieves the sum of the two factors, it is considered an acceptable value (39–41). The first factor, which is named health literacy, has a value of 47.42%, which indicates that the first factor items significantly affect how people are informed and break the disease's transmission chain. The second factor is “home health empowerment,” which, according to the results, has a value of 18.62%. Based on this factor, reducing and controlling this disease occurs when people's knowledge about the prevention of the disease affects their attitude toward high-risk family members, which is the best method to prevent other peoples from becoming infected.

In the items related to the factors, we are faced with items about pets in the first factor. According to Iran's cultural conditions, people with pets are considered a minority in society, and we see this in the results obtained in the Lorink factor. Pet (dogs and cats) hygiene in the COVID-19 era is vital because recent studies have indicated that the virus could be transmitted from pets to humans and *vice versa*. So it is crucial to having protective attitudes that keep both humans and pets distanced, to prevent becoming infected and to prevent further transmissions of the virus (42).

Hence, based on EFA, the final version of KA-C is considered a two-factor scale with construct validity. In this analysis, the second-order CFA was performed to explore a more detailed model of structural equations. This approach aims to obtain a more important data collection method, assuming that one or more higher-order factors are responsible for the latent variables in the common variance and that the proposed scale has two orders (43). A high correlation between the first constructs shows that the latent variables do not behave as a variable that is entirely independent. At the secondary conceptual level, the connection between them indicates a more general framework where structural equation modeling is the best tool for structural assessment because it can classify first-order structures that have been suggested as latent variables (44).

Furthermore, the most common indicators of goodness-of-fit were evaluated. Based on the results, goodness-of-fit for all indicators was appropriate. Some research has indicated that by first-order factor analysis, the intended construct must first be developed. The conceptual construct's excellent fit is then calculated using the second-order factor analysis to test the structural equation model (45).

Cronbach's alpha correlation coefficient, omega McDonald and AIC of factors was an acceptable internal consistency. Internal consistency is usually a measure by which it tests the correlation of different items in a test (or similar subscales in a larger test). It also examines whether several items that measure the same overall structure produce identical scores (46).

SARS-CoV-2 is a new and extremely infectious virus, and there is currently no precise treatment for COVID-19. It must be considered that if no appropriate action is taken, and if medications, vaccines, and patient monitoring programs are not widely enforced or effective, preventive behaviors are likely to continue until 2022 (47, 48). Currently, as vaccinations are underway, the extent and duration of immunity to SARS-CoV-2 remain poorly understood therefore still need to emphasize preventive measures. In addition to preventive measures, critical care capacities should also be expanded to heighten preventive measures' success (48). So, scales that provide an overview of the community knowledge and attitudes toward the prevention of COVID-19 are practical in disaster management at this time.

Altogether, the primary intervention in disaster management is based on proper information about “one health” and attitudes to this information, to best control this pandemic. Also, health policymakers could benefit from this scale—by assessing the people's knowledge and attitudes, they may gain better insights on their policy making.

## Limitation

One limitation of this study is that this research was only performed in Iran, despite the appropriate number of samples. Since it was mentioned that cultural factors might also affect the results, it is better to also include other countries and cultures to prove its validity and reliability. Due to the disease's current situation, the construction and distribution of this scale were done electronically; thus, there was a decreased opportunity to send it to lower economic status groups who do not have access to cyberspace. Moreover, the mentioned economic issues also affect the results.

## Conclusion

It is crucial to attract attention toward factors that engage people's knowledge and attitudes toward COVID-19 and how to break the transmission chain using the Persian version of the KA-C scale. We designed and developed a scale using multifaceted variables through this study, operationalizing them on the validated scale. The final scale consisted of 18 items and two factors that explained 66.05% of the total variance. This scale could bring robust suggestions for health policies toward COVID-19 transmission control across the world. Furthermore, the factors of “health literacy” and “home health empowerment” are a new basis for capturing such infrastructures' interaction in providing important information for focused health policies. Altogether COVID-19 prevention, with different transmission methods, requires collaboration among different sectors, public and governmental institutions.

## Data Availability Statement

The data sets generated for this study are available at reasonable request to the corresponding author.

## Ethics Statement

The studies involving human participants were reviewed and approved by This study was approved by the Ethics Committee of Mazandaran University of Medical Sciences by the code of IR.MAZUMS.REC.1399.596. All participants voluntarily participated in this study. Names of the participants were not mentioned in the study, and instead of their names, codes were used in the interview texts. The participants' statements were not discussed with other research team members to maintain the information's confidentiality. Also, in the initial set of data gathering, participants were ensured that all answeres remain confidential, and involvement in the study is voluntary. Written informed consent to participate in this study was provided by the participants' legal guardian/next of kin.

## Author Contributions

All authors contributed to the study conception and design. Material preparation, data collection performed by MA, MM, MS, ShagK, and ShahK. PR and HS analyze data. The first draft of the manuscript was written by MA, MM, PR, ShagK, and ShahK and all authors commented on previous versions of the manuscript. All authors read and approved the final manuscript.

## Conflict of Interest

The authors declare that the research was conducted in the absence of any commercial or financial relationships that could be construed as a potential conflict of interest.
